# A novel multi-scale CNN and Bi-LSTM arbitration dense network model for low-rate DDoS attack detection

**DOI:** 10.1038/s41598-024-55814-y

**Published:** 2024-03-01

**Authors:** Xiaochun Yin, Wei Fang, Zengguang Liu, Deyong Liu

**Affiliations:** 1 Shandong Provincial University Laboratory for Protected Horticulture, Weifang Key Laboratory of Blockchain on Agricultural Vegetables, Weifang University of Science and Technology, Weifang, 262700 China; 2 Computer Science and Engineering College, Weifang University of Science and Technology, Weifang, 262700 China; 3 School of Information Engineering, Shandong Vocational College of Science and Technology, Weifang, 261053 China

**Keywords:** Arbitration mechanism, Dense connection, Embedding-based bi-LSTM, LDDoS attacks, Multi-scale CNN, Network security, Computational science, Computer science

## Abstract

Low-rate distributed denial of service attacks, as known as LDDoS attacks, pose the notorious security risks in cloud computing network. They overload the cloud servers and degrade network service quality with the stealthy strategy. Furthermore, this kind of small ratio and pulse-like abnormal traffic leads to a serious data scale problem. As a result, the existing models for detecting minority and adversary LDDoS attacks are insufficient in both detection accuracy and time consumption. This paper proposes a novel multi-scale Convolutional Neural Networks (CNN) and bidirectional Long-short Term Memory (bi-LSTM) arbitration dense network model (called MSCBL-ADN) for learning and detecting LDDoS attack behaviors under the condition of limited dataset and time consumption. The MSCBL-ADN incorporates CNN for preliminary spatial feature extraction and embedding-based bi-LSTM for time relationship extraction. And then, it employs arbitration network to re-weigh feature importance for higher accuracy. At last, it uses 2-block dense connection network to perform final classification. The experimental results conducted on popular ISCX-2016-SlowDos dataset have demonstrated that the proposed MSCBL-ADN model has a significant improvement with high detection accuracy and superior time performance over the state-of-the-art models.

## Introduction

With the widespread deployment of cloud computing network, LDDoS attacks have been reported as the notorious network security issues. These attacks are a form of periodic multi-variate time series pulse flow where a malicious user floods the cloud service stealthily. They can severely degrade the quality of service confirmed by researchers^1^. But their behaviors are alike to normal flows in terms of speed and volume, making them difficult to distinguish from normal flows. For example, the BlackNurse attack reported by TDC Security Operations Center^2^ could degrade operators’ cloud services with the traffic speed of 5 packets per second, and offline operators’ firewalls if the traffic volume bumped to 15–18 Mbit/s. Thus, LDDoS attacks attract more and more attentions, and many LDDoS variants with smarter behaviors are designed. Hivenets^3^ used bee colony algorithm to guide a group of intelligent bots to fire autonomous LDDoS attacks with minimal supervision. A multi-target LDDoS attack model^4^ tried to utilize and orchestrate bots’ spare time slots between bursts to fire new LDDoS attacks. And the bad news is these smarter LDDoS attacks are more strenuous to be captured. Even worse, all the traffics in the network are impacted heavily at the same time, which leads to packet loss to some extent. This is why the big-scale public LDDoS dataset is scarce. As a result, designing and training a powerful and satisfactory LDDoS attack detection model based on the limited poor data is a changeling task.

Even the performance of models is positively correlated with the dataset, researchers tried to centralize datasets that are scattered in different clients to train a powerful model with high performance. Federated Learning (FL) is just this kind of paradigm offering joint learning through multiple datasets. Wang et al.^5^ proposed an intrusion detection method based on FL and CNNs to solve the problem of training a depth model with high accuracy under the limited labelled data generated by a single mechanism. Li et al.^6^ proposed a novel DeepFed federated deep learning framework. The framework employed CNN and gated recurrent unit (GRU) as the basic layers of intrusion detection model, and allowed multiple clients to collectively train the parameters of model. The experiments showed the proposed DeepFed scheme had the high effectiveness in detecting network intrusion. Mothukuri et al.^7^ proposed a GRU-based IoT networks intrusion detection model. The weights of central model were updated from multiple clients to optimize its accuracy by federated training rounds. This method kept the data intact on local IoT devices by sharing only the learned weights and guaranteed the data safe. Idrissi et al.^8^ proposed an autoencoder-based method for distributed network intrusion detection systems by leveraging FL and anomaly detection. The method computed an intrusion score based on the reconstruction error while preserving the privacy of local data. Wu et al.^9^ proposed a novel LDDoS attack detection FL framework using transfer learning and support vector machine (SVM). The framework performed aggregation of data distributed in various clients through FL, then utilized transfer learning and SVM build personalized models. Bertoli et al.^10^ introduced the stacked-unsupervised FL approach for detecting a flow-based network intrusion. The novel method comprised a deep autoencoder in conjunction with an energy flow classifier in an ensemble learning task. These methods are available to use the high-quality LDDoS attack examples in different data nodes. However, their deep learning models are insufficiency to learn time sequence features. Thus, Tang et al.^11^ used Gramian angular summation field transformation to analyze time-based features for finding out the attack, and the results were effective. With the emerging LSTM, researchers integrated it into FL framework. By taking advantage of LSTM, Zhao et al.^12^ proposed an effective intelligent intrusion detection method based on FL aided LSTM framework, which can solve the problem of a powerful deep learning model training and intrusion risks at the central server and violate user privacy if collecting dataset from all the user servers. Huong et al.^13^ proposed the hybrid variational autoencoder-based LSTM model based on FL for time series data anomalies. The architecture achieves high detection performance in time series data anomalies. Zhang et al.^14^ proposed a vertical federated learning framework based on LSTM fault classification network. The framework could optimize model parameters on the entire firefighting IoT platform, and get the effective predicted results. Wang et al.^15^ designed an attention-based bi-LSTM model within the framework of multi-domain FL framework for detecting coordinated network attacks. Liu et al.^16^ proposed an asynchronous FL arbitration framework named AsyncFL-bLAM based on bi-LSTM and attention model. The novel arbitration detection model took on the responsibility of LDDoS attack detection locally.

The above methods can use well-labelled training data across many organizations. However, when researchers examined and compared the performance of LDDoS attack detection methods deployed by centralized mode and FL mode^17^. The accuracy and outperforming of models in FL mode could only close to, but lower than ones in centralized mode. Thus, researchers turned to LDDoS data enhancement and novel model design. At early stage, Random Forest (RF)^18^, SVM^19^, K-Near Neighbor (KNN)^20^ and their enhanced shallow models with well-designed feature engineer were adopted for LDDoS attack detection. Tang et al.^21^ proposed performance and features framework for LDDoS attacks based on SVM, KNN and so on. The framework analyzed the performance of normal traffic and made full use of flow features, which gained a high detection accuracy. Tang et al.^22^ also enhanced the fine-grained detection model by adaptive Kohonen network cluster analysis algorithm. As a result, the proposal had an ability to accurate fine-grained detection and detect every attack burst. Muhammad et al.^23^ proposed a multivariate chart by integrating support vector data description and kernel density estimation. The chart can monitor the network’s anomaly with high performance. Later, researchers realized that deep learning models were superior to shallow ones in detection accuracy and scalability. Liu et al.^4^ introduced locality sensitive features extraction method for classifying the different flows into small buckets ahead, and then proposed a simple 3-layer CNN-based model to extract key feature representations. Zhou et al.^24^ proposed a few-shot learning model with siamese CNN (FSL-SCNN) to alleviate the over-fitting issue and enhance the performance for intelligent anomaly detection. The FSL-SCNN encoding network was helpful to optimize feature representations and enhance the efficiency of the training process. Asgharzadeh et al.^25^ worked out CNN-BMECapSA-RF model based on CNN and hybrid layers to enhance classify accuracy, and developed a binary multi-objective enhanced capuchin search algorithm to implement feature extraction automatically. Ren et al.^26^ proposed a novel CANET network. The CNN network and attention mechanism are mixed for local spatio-temporal feature extraction of LDDoS attacks. Experiments demonstrated CANET is efficient in accuracy, detection rate, and false positive rate. Venkateshwarlu et al.^27^ proposed a framework named LRDADF, which used CNN and deep sparse autoencoder to detect LDDoS attack. The sparse autoencoder was used to learn from traffic patterns and CNN was for classifying. Considering the time series properties of LDDoS, Salahuddin et al.^28^ proposed an anomaly detection system based on autoencoder to leverage time-based features over multiple time windows for efficiently detecting anomalous LDDoS. Romany^29^ employed adaptive harmony search algorithm for feature selection, and attention based bi-directional gated recurrent neural network model for time-based feature classification. And researchers introduced LSTM network in LDDoS attack detection, since its good performance in time sequence data. Zhou et al.^30^ designed a variational LSTM (VLSTM) deep model for IoT attack detection. It used encoder-decoder network to reconstruct feature representation, used a variational reparameterization scheme to learn key feature representation, and used three loss functions to control weigh learning. Mushtaq et al.^31^ proposed a hybrid framework comprising deep auto-encoder with LSTM and bi-LSTM for attack detection. In this framework, the auto-encoder was used to obtain optimal features and LSTMs were used to finish the classification task. Liu et al.^32^ proposed a novel, practical and fast ensemble model FastCBLA-EM. It used a competitive network composed of 1-D CNN and a bi-LSTM network to learn feature representations of samples, and then, an arbitration mechanism was used to weigh the representations for classifying LDDoS attacks. Du et al.^33^ proposed NIDS-CNNLSTM model for network security. It combined the powerful learning ability of LSTM network in time series data to extract key features, and use CNN network to classify those filtered features.

To abridge, the state-of-the-art centralized methods took use of both CNN and LSTM for feature extraction. But these existing methods used CNN and LSTM sequentially. The CNN was used to extract internal features, and then, the LSTM was used to extract association based on the output of CNN. This means it is possible to loss important spatio-temporal features. This is unbearable on the limited poor dataset, specially. Thus, in this paper, we propose a novel multi-scale CNN and bi-LSTM arbitration dense network model MSCBL-ADN for alleviating the impact of the insufficiency of high-quality attack examples. The contributions of this paper are as follows:

- The paper introduces a novel fixed *T*-length sliding segmentation data augmentation method to split raw multi-variate data into multiple equal length detection blocks real time. This not only enlarges the high-quality attack examples, but also speeds up the LDDoS attack detection.
- The paper proposes a MSCBL-ADN model, which incorporates multi-scale CNN for preliminary spatial feature extraction and embedding-based bi-LSTM for time relationship extraction; which employs arbitration network for high accuracy by redistribution of the weights of key representations and uses 2-block dense connection network to perform final classification.
- The paper compares MSCBL-ADN model with state-of-the-art models by classification accuracy and time consumption on ISCX-2016-SlowDos dataset. The experimental results demonstrate the effectiveness of the proposed method.

The rest of the paper is organized as follows: In Section “Methods”, the data augmentation method and the architecture of MSCBL-ADN model have been expressed in detail. In Section “Results”, the process of ISCX-2016-SlowDos dataset augmentation and the experiment environment are introduced. And the performance evaluation is described in terms of the detection accuracy and the time consumption. “Discussion” Section covers the analysis of the ablation experiments and contrast experiments. Finally, in Section “Conclusions”, the paper is concluded and suggests future directions briefly.

## Methods

**Figure 1:**
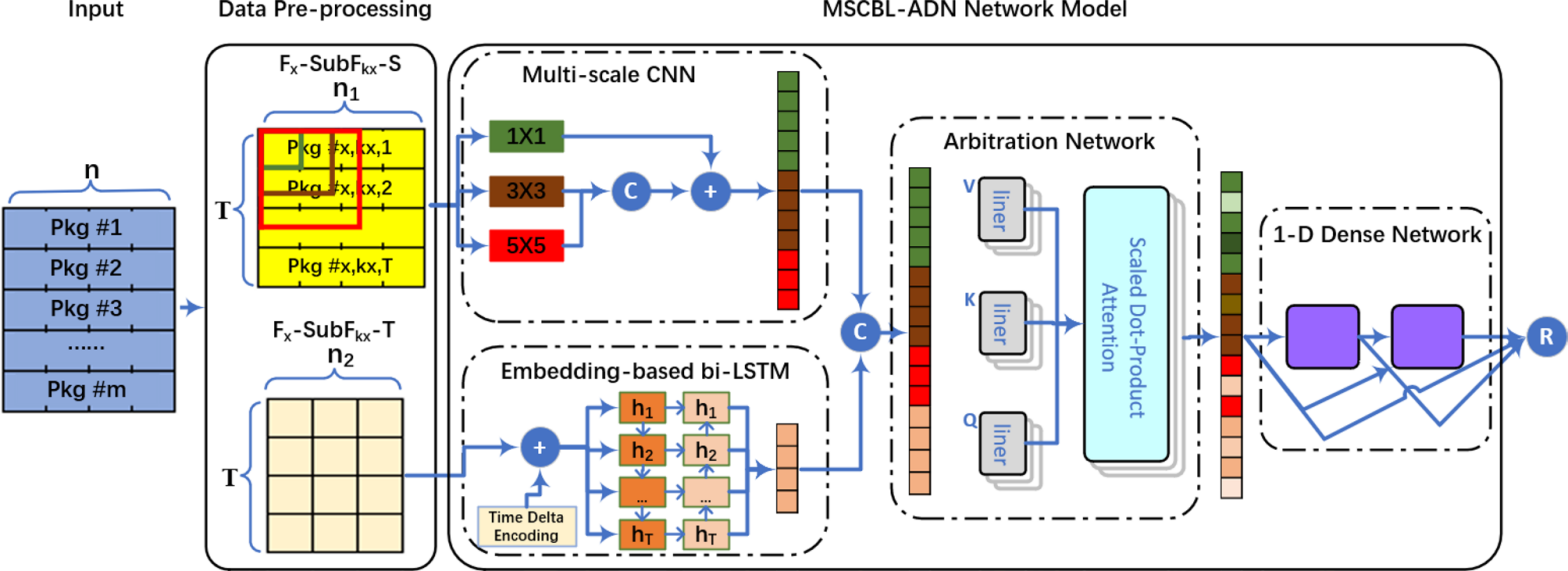
Block diagram of the proposed methodology.

A novel multi-scale CNN and bi-LSTM arbitration dense network model is proposed and explained detailed in this section. As illustrated in Fig. 1, the block diagram includes data pre-processing and MSCBL-ADN network model. In data pre-processing part, the captured packets are organized into different flows per their flow 5-tuple (source IP, source port, protocol, destination IP, destination port). And then, the flow is segmented into multiple detection blocks by the default length *T*, named $F_x$-$SubF_{kx}$. The feature number in $F_x$-$SubF_{kx}$ is *n*. Since the raw multi-variate data contains spatio-temporal features, the $F_x$-$SubF_{kx}$ is split into $F_x$-$SubF_{kx}$-*S* for spatial feature set and $F_x$-$SubF_{kx}$-*T* for time relationship feature set, whose numbers are $n_1$ and $n_2$ separately. In MSCBL-ADN network model part, the spatial features are handled by multi-scale CNN network with $1 \times 1$, $3 \times 3$ and $5 \times 5$ convolutional kernels. The extracted features are concatenated and flattened into a vector. Parallelly, the bi-LSTM networks are used to learn the valid information from time delta embedding $F_x$-$SubF_{kx}$-*T* dataset through forward and backward propagation. After that, the learnt temporal representation vector concatenates with spatial feature vector. And then, an arbitration network is adopted to redistribute weights by multi-head attention mechanism. As shown, the critical representations are given heavy weights. At last, 1-D dense network with two dense blocks is employed for classifying. Overall, some important methods, such as batch normalization, *softmax* function, cross-entropy loss and dropout technology, are used in MSCBL-ADN model for eliminating over-fitting problem.

### Fixed *T*-length sliding segmentation method

**Figure 2:**
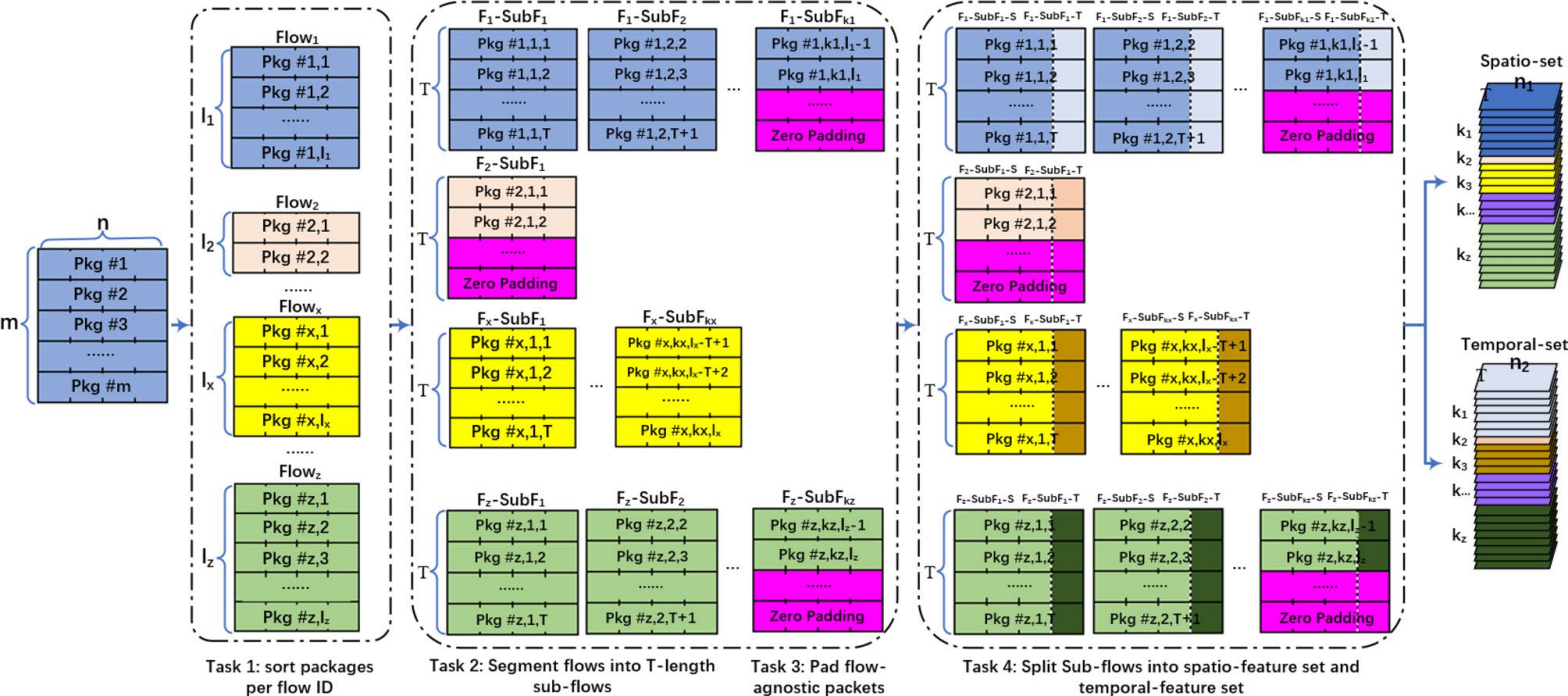
Fixed *T*-length sliding segmentation method.

The MSCBL-ADN model needs to be trained based on the limited poor LDDoS dataset, and run in real-time network. For that, the paper introduces a novel fixed *T*-length sliding segmentation method for both data augmentation and speeding detection up. The method is proposed as illustrated in Fig. 2. It mainly has four tasks. One of tasks is to sort packets and extract features to form flows based on communication quintuple and arrival time. Sequentially, the second task is to segment flows into fixed *T*-length sub-flows. And then, the third task is to pad the flow-agnostic packets, once the length of the last sub-flow is less than the required length during training phase or the expected packets don’t arrive yet. Finally, the method splits multi-variate sub-flows into spatial part $F_x$-$SubF_{kx}$-*S* and time relationship part $F_x$-$SubF_{kx}$-*T*. As a result, the high-quality LDDoS training examples are augmented and the LDDoS attack detection is speeded up.

In task one, we find that the collected packets on dataset and in real network world are mixed up and sorted by the arrival time. The reason is that the physical links are shared and multiplexed by flows. But the network attacks fire from fixed victims normally. This asks us to distinguish flows. Therefore, we sort packets based on communication quintuple and arrival time. Let the number of packets be *m*, the number of flows be *z*, then $m=\sum _{i=1}^zl_i$, where $l_i$ means the length of $i^{th}$ flow. Next in task two, the sliding window with the fixed *T*-length is used to segment LDDoS attack detection blocks on the flows. By default, the stride of sliding window is set to 1 for data augmentation purpose. Thus, we will have a new sub-flow detection once a new packet is arrived. Take sub-flows $F_1$-$SubF_1$ as example, when new packet “Pkg #1,2,*T*+1” is arrived, the new *T*-length detection block $F_1$-$SubF_2$ is formed. Its *T*-1 packets are same with $F_1$-$SubF_1$. By this method, the MSCBL-ADN model can detect whether LDDoS attack happens when any new packet arrives in real network world. It is obviously that the detection speed is accelerated. As to task three, the method helps to dilate the sub-flows with zero-padding packets, when the number of packets in the sub-flow is less than *T*. Take $F_2$-$SubF_1$ as examples, there are only a few packets collected when LDDoS attack detection is triggered, since it is a new sub-flow. It is obviously that the count of collected packets is less than *T*. For the input requirement of MSCBL-ADN model, we must dilate the sub-flow. Take $F_z$-$SubF_{kz}$ as examples, its last packet is agnostic. That is, the count of packages in the last segment may be less than *T*. For that, we must pad the sub-flow. When reviewing the MSCBL-ADN model, there is a bi-LSTM network part. It is high time consumption, especially when huge feature set is fed into. But these time-independent features have almost no impact for detection accuracy^32^. Therefore, in task four, we split the original multi-variate sub-flows into spatial feature sub-flows $F_x$-$SubF_{kx}$-*S* and time relationship sub-flows $F_x$-$SubF_{kx}$-*T*. After above operations, the raw data are reshaped into the tensor of $\sum _{k=1}^zk_i*(n_1+n_2)*T$ as shown in Eq. (1),

1 $$\begin{aligned} k_i= {\left\{ \begin{array}{ll} l_i-T+1 &{} l_i \ge T \\ 1 &{} l_i < T \end{array}\right. }, \end{aligned}$$

where $k_i$ is the number of segmentations to be trained/detected, $n_1$ and $n_2$ are the number of spatial and temporal features separately. And we label the corresponding sub-flows per its original flow classification in training set.

Obviously, the method has the several advantages. First, it can speed the frequency of detection up. Once any new package arrives, the detection can start without waiting for all the packages on this flow. Second, multi-variate data is split and fed into CNN and bi-LSTM paths parallelly, which save time consumption further.

### Multi-scale CNN network

**Figure 3:**
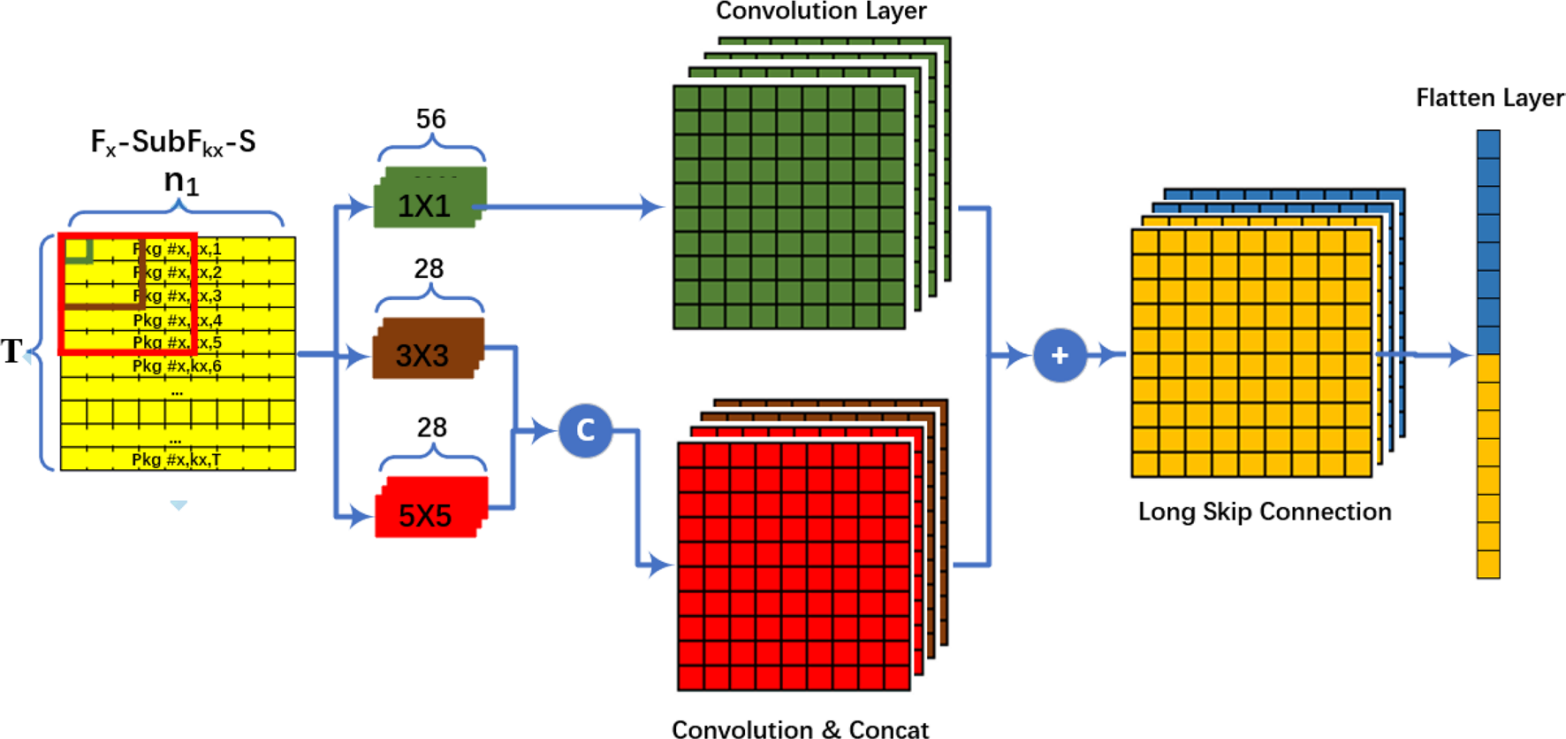
Framework of the multi-scale CNN network.

LDDoS attacks have the characteristic of periodicity. As shown in Fig. 3, the multi-scale CNN network is designed to handle the insufficient utilization of LDDoS attack packet. It adopts a multi-scale feature fusion extraction channel that combines skip connections and feature fusion operations. Usually, this kind of periodicity can be analyzed by observing 3–5 packets at least. Take slow-body attacks as examples, they continue to send small data packets to server with an ultra-slow speed once the connects are established. The first packets in these attacks have bigger content-length values in their HTTP headers normally. And the sequential data packets have the almost same HTTP header, but different HTTP body. Thus, we need to analyze 3–5 packets before making a decision. The 5$\times$5 kernel is just used for finishing this kind of analysis. Another LDDoS attacks, such as slow-headers, aim to deplete CPU resources or network connection pools. They attack servers with endless HTTP headers. These packets have same HTTP headers and can be analyzed through 1–3 packets. The $3 \times 3$ kernel is good at handing these cases. The $1 \times 1$ kernel helps to add non-linear into the original data and ascend dimensions. This improves the presentation ability of the multi-scale CNN network. After above convolutional operations, these extracted presentations are fused and flatted by long skip connections for prediction.

Firstly, three convolutional kernels of different sizes, 1$\times$1, 3$\times$3 and 5$\times$5, are used to extract features from detection blocks. The 1$\times$1 convolutional kernel in the first channel is used to obtain detailed features of network packets. In this channel, we use 56 1$\times$1 convolutional kernels. The formula is shown in Eq. (2),

2 $$\begin{aligned} F_1(Y)=W_1 \times F_x\text {-}SubF_{kx}\text {-}S + B_1 \end{aligned}$$

where $F_1(Y)$ is the outputted feature map by the first channel. $W_1$ is the weights of 56 1$\times$1 convolutional kernels and $B_1$ represents the bias. Normally, LDDoS attack flows are periodic multi-variate time series pulses. That is, the packets nearby have strong positive correlation. Thus, we use 28 3$\times$3 convolutional kernels in the second channel and 28 5$\times$5 convolutional kernels in the third channel. They are used to cope with spatial relationship among packets. The formula is shown in Eq. (3),

3 $$\begin{aligned} \begin{aligned} {\begin{matrix} &{}F_2(Y)=W_2 \times F_x\text {-}SubF_{kx}\text {-}S + B_2, \\ &{}F_3(Y)=W_3 \times F_x\text {-}SubF_{kx}\text {-}S + B_3 \end{matrix}} \end{aligned} \end{aligned}$$

where $F_2(Y)$ and $F_3(Y)$ are the outputted feature map of the second channel and third channel separately. $W_2$ and $W_3$ are the weights of all those 3$\times$3 and 5$\times$5 convolutional kernels. $B_2$ and $B_3$ represents the bias.

And then, a *Concat* operation is adopted to combine the different scales of feature information after the second and third channels. At the same time, we use the long skip connections to fuse the multi-scale feature information. This ensures the completeness of LDDoS attack features from detection blocks, and thereby improving the ability to extract useful high-frequency spatial information. The formula is shown in Eq. (4),

4 $$\begin{aligned} \begin{aligned} {\begin{matrix} &{}F_{23}(Y)=Concat(F_2(Y) + F_3(Y)), \\ &{}F(Y)=W \times (F_1(Y) + F_{23}(Y)) + B \end{matrix}} \end{aligned} \end{aligned}$$

where $F_{23}(Y)$ is the concatenated feature map of the second channel and third channel, the size is $(56, T, n_1)$. And the size of $F_1(Y)$ is $(56, T, n_1)$, too. Thus, we add $F_{1}(Y)$ and $F_{23}(Y)$, and get *F*(*Y*). *W* is the weights and *B* is the bias in this fusion layer.

At last, a *Flatten* layer is for transforming multi-dimensional feature maps into one-dimensional arrays for next arbitration network.

### Time delta embedding-based bi-LSTM Network

**Figure 4:**
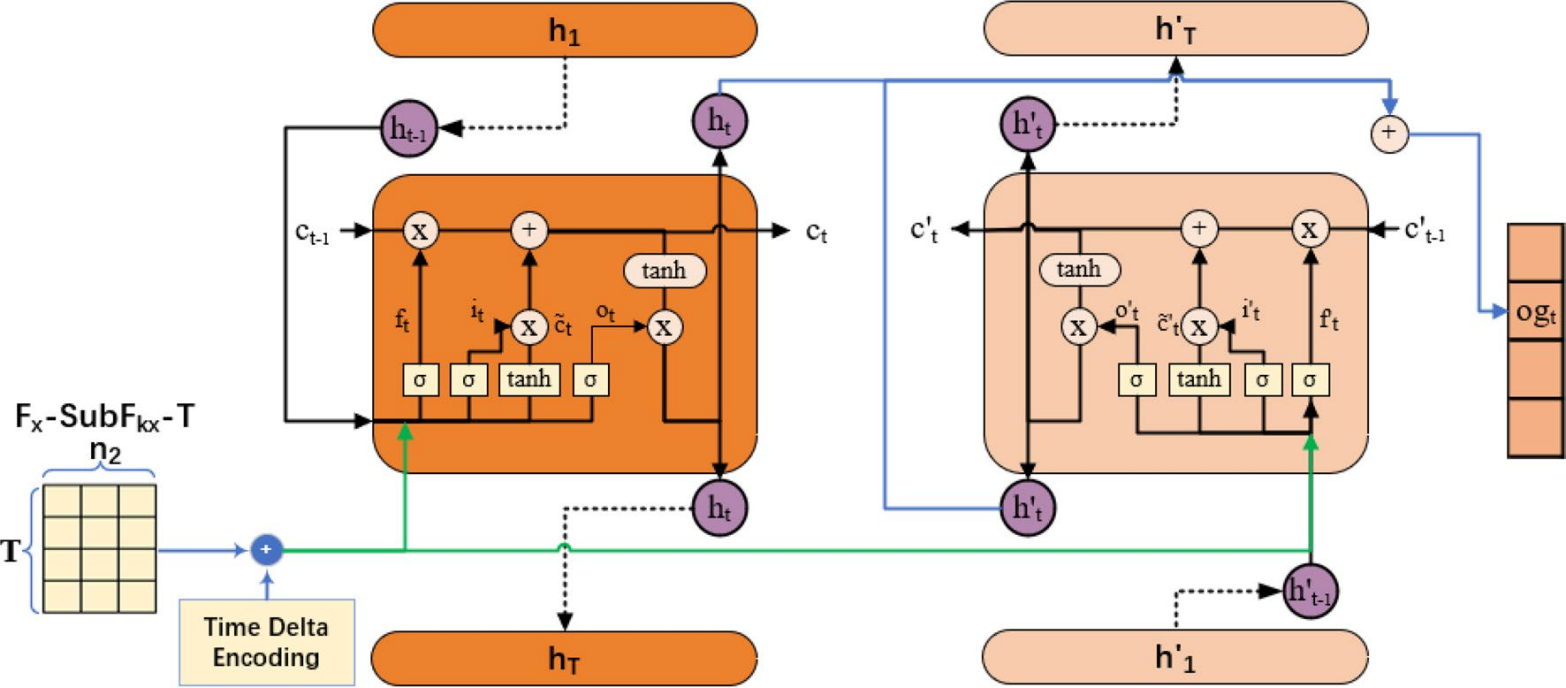
Framework of time delta embedding-based bi-LSTM network.

The collected LDDoS attack sub-flows are composed of a sequence of attack packets. They are seen as time series data. Thus, they can be coped with LSTM for time relationship information. But there may be some packets missed in detection blocks under the condition of network jam in our case. We need refer whether LDDoS attacks happen from forwards and backwards directions simultaneously. Naturally, bi-LSTM network, which uses stacked two LSTM layers, is the effective mean as shown in Fig. 4. It can learn long-term and short-term time relationship information of network packets from both directions. Nevertheless, LSTM-like network is time-consuming. And the consumed time mainly comes from two aspects. The first aspect is introduced by its recurrent structure. As known, the calculation of current time step must wait the hidden variables in last time step in LSTM-like network. As a result, the calculations in different time steps are hard to execute parallelly. Thus, we turn to the second aspect, the computation. Per our research, too many inputs mean large computations. we propose a time-delta embedding method through encoding and fusing limited key time-related features as input to get hidden variables. These features include ‘arrival time’, ‘begin time’ and ‘time delta’ so on. The ‘arrival time’ is the key feature, which includes the chronological order of LDDoS attack. The ‘time to live’ means how many networks the packet has passed through over time. It can be used to infer the ‘begin time’ of attacks. Most importantly, we introduce the ‘time delta’ feature between any two packets in same sub-flow. It is an import indicator of LDDoS attack, which can indicate whether flow is similar. As the input features decrease, the computational workload greatly cuts down.

The import components of bi-LSTM are LSTM memory cell and bidirectional self-cycling mechanism. The LSTM memory cell adopts the forget gate $f_t$, input gate $i_t$ and output gate $o_t$. The $f_t$ determines what is essential to retain from the previous state, the $i_t$ determines how to add new information to the cell state, and the $o_t$ defines what should be the next hidden state. In addition, $\tilde{C}_t$ represents the candidate updating value. Thus, the outputs of current LSTM cell $h_t$ and $h'_t$ can be calculated through Eq. (5),

5 $$\begin{aligned} \begin{aligned} {\begin{matrix} &{}h_t=\sigma (W_ox_t+U_oh_{t-1}+b_o) \times tanh(\sigma (W_fx_t+U_fh_{t-1}+b_f)C_{t-1}+\sigma (W_ix_t+U_ih_{t-1}+b_i)\tilde{C}_{t-1}),\\ &{}h'_t=\sigma (W'_ox_t+U'_oh'_{t-1}+b'_o) \times tanh(\sigma (W'_fx_t+U'_fh_{t-1}+b'_f)C'_{t-1}+\sigma (W'_ix_t+U'_ih_{t-1}+b'_i)\tilde{C'}_{t-1}). \end{matrix}} \end{aligned} \end{aligned}$$

where $x_t$ is the input at time *t*. $W's$, $U's$ and $b's$ denote the weights and bias of $f_t$, $i_t$, $o_t$. *sigma* and *tanh* are activation functions, while $\times$ means dot multiplication. And then, the $og_t$ can be gotten by Eq. (6),

6 $$\begin{aligned} og_t=W_4h_t+W_5h'_t. \end{aligned}$$

and it has bi-directional feature representations.

### Multi-head attention arbitration network

**Figure 5:**
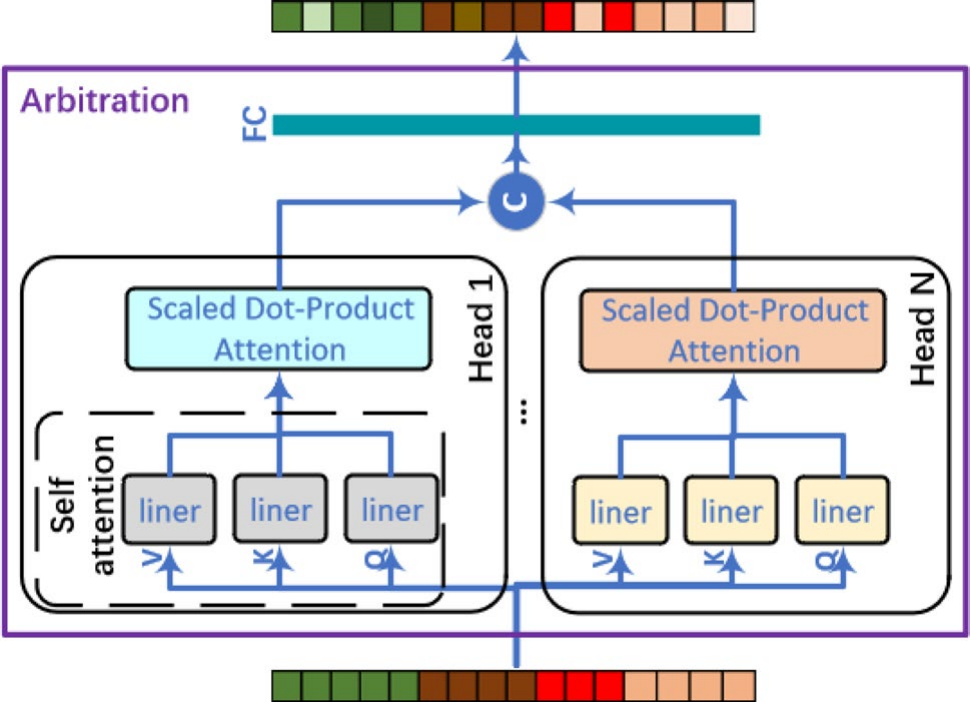
Framework of multi-head attention arbitration network.

The extracted features by multi-scale CNN and bi-LSTM are contacted into a vector. And the feature vector is fed into the arbitration network for their weights redistribution. The arbitration network depends mainly on multi-head attention mechanism to efficiently extract information from the input feature vector, as depicted in Fig. 5. Firstly, the arbitration network projects at the *Qs*, *Ks*, and *Vs* *N* times with different linear models. They are self-attended, that is, the projected *Qs*, *Ks*, and *Vs* come from that same extracted feature vector. Then, the *Qs*, *Ks*, and *Vs* feed into scaled dot-product attention module in parallel, and yield result values by Eq. (7),

7 $$\begin{aligned} Head_i=Softmax\left( \frac{QK^T}{\sqrt{d}}\right) V. \end{aligned}$$

where $Head_i$ is the result value of head *i* ($i<N$). *d* represents the dimensional of *Qs* or *Ks*. At last, the result values are concatenated and projected once again, resulting in the arbitration values by Eq. (8),

8 $$\begin{aligned} arbitration=FC(Concat(head_1, \cdots , head_N)). \end{aligned}$$

After this arbitration network, the upstream extracted features by different network models are weighed. It helps find the important features out. And it has strong parallelism capability for speeding LDDoS detection up.

### 1-D dense classification network

To further keep the key LDDoS detection feature representation between layers, the direct connections from any layer to its subsequent layers are adopted in a feed-forward fashion. That is, the multiple output vectors produced in layers from 0 to $l-1$ are concatenated into a single tensor as described in Eq. (9),

9 $$\begin{aligned} x_l= Concat([x_0, x_1, \cdots , x_{l-1}]). \end{aligned}$$

where $x_i$ refers to the output vector in layer *i*. There are two dense blocks in our proposal. And we add $2\times 2$ average pooling operation between blocks, which can down-sample and shrink the size of feature vectors. In this 1-D dense network, there are many different paths between layers, which can alleviate the vanishing-gradient problem effectively. At the same time, the convolutional kernels can be reduced since the feature is propagated well between layers. This means the number of parameters in model substantially reduces, which can speed LDDoS detection up further.

For getting the classification of LDDoS, *softmax* activations are used in last layer. And during MSCBL-ADN model compiling, $categorical\_crossentropy$ loss function is used to calculate cost in training set and validation set. *Adam* optimizer is used to adjust weights and biases through back propagation.

## Results

In this section, the dataset, experimental environment and results are given. Firstly, the public ISCX-2016-SlowDos dataset is descripted and revised for the sequenced experiments. And then, experimental environment is deployed, hyperparameters are listed, and evaluation indicators are descripted. At last, experiments and comparable experiments are carried out in terms of accuracy and computational time complexity.

### Dataset description, revision and pre-processing

To verify the detection accuracy and time performance of MSCBL-ADN model, a reliable, multi-class, and small-scale balanced raw-format dataset is needed. The popular low-rate DDoS detection dataset ISCX-2016-SlowDos^34^, provided by Canadian Institute for Cybersecurity, is collected from the dynamic, real and complex network testbed and stored as the raw-format data. This declares the reliability of these collected packets without doubt. In terms of multi-class labeling, the dataset includes different kinds of DDoS packets, such as low-rate and high-volume ones, but the bad news is that the packets are all labelled as single malign type. Even worse, there is not any benign packet captured on the dataset. In this point, the dataset without revision is not suitable for verifying multi-class detection ability of MSCBL-ADN model. The other reason for revising the ISCX-2016-SlowDos dataset is to validate the model’s learning ability on small-scale dataset. As described in above sections, the novel LDDoS attacks are increasingly hard to be captured. As a result, the size of the training dataset is also decreasing reasonably. To simulate this situation, we need to perform down-sampling on the original dataset.

Based on above principles and rationalities, we firstly filter RUDY, slow-body, slow-headers, and slow-read LDDoS attacks out. Taking RUDY as an example, its target servers are “208.113.162.153” and some other listed IP’s per the official documents^34^. Thus, we apply the filter of “ip.src==208.113.162.153 or ip.dst==208.113.162.153” to filter raw RUDY network packets out in WireShark tool. The same method is applied to other IP’s and other LDDoS attack type. And then, we keep hulk attack on this dataset as high-volume DDoS attack and choose packets from 1999 DARPA dataset^35^ as benign ones for the purpose of multi-class detection. The training data of first week on 1999 DARPA dataset does not contain any attacks^35^. And the packets are stored as raw-format data, which are consistent with the ones on the ISCX-2016-SlowDos dataset. They can be intermixed easily later. Next, we sort the packets by communication quintuple and segment them by TCP SYN, FIN, and RESET flags into flows. At last, a random counter and a packet counter are used for generating the revised dataset. By the random counter, a certain flow is selected. By the packet counter, the data down-sampling is performed, and the size of dataset is under control. When we set the packet counter for every attack, a small-scale revised dataset is gotten. The detail of the revised dataset is shown in Table 1.

**Table 1:** Summary of characteristics on the revised ISCX-2016-SlowDos datasets.

DoS attack type	No. of flows	Av. flow size (pkt)	Av. duration (s)
RUDY	2268	8	38.41
Slow-body	615	15	834.01
Slow-headers	3672	7	45.93
Slow-read	226	31	153.91
Hulk (High-volume DDoS)	38	118	44.95
benign	800	23	2.27

After getting the revised dataset, the packets in flows are pre-processed. The one-hot encoding is applied to transform the categorical features into numeric features, such as protocol type, and the max-min normalization is performed for the purpose of preventing gradient explosion during model training. To learn patterns of flows, the flows are further split into sub-flows (samples) and labeled by the fixed *T*-length sliding segmentation method. The detailed operation can refer to section Methods. In this way, the conventional packet-based features are formed to many fixed length two-dimensional sub-flows, by which the model can learn LDDoS attack patterns based on current and previous $(T-1)$ packets. The size of sub-flows is with 85.71% overlap, since the default stride is set to 1. As known, the detailed experimental results of MSCBL-ADN model are discussed on both binary-class and multi-class scenarios. In the binary-class scenario, we label both hulk and benign samples as normal classification, and label others as LDDoS classification. In multi-class scenarios, the attacks keep their original labels. At last, the dataset is randomly partitioned into two small sets by 80%:20% by using the train_test_split() function of the sklearn library. The 80% dataset is for training, while the 20% one is for testing.

**Figure 6:**
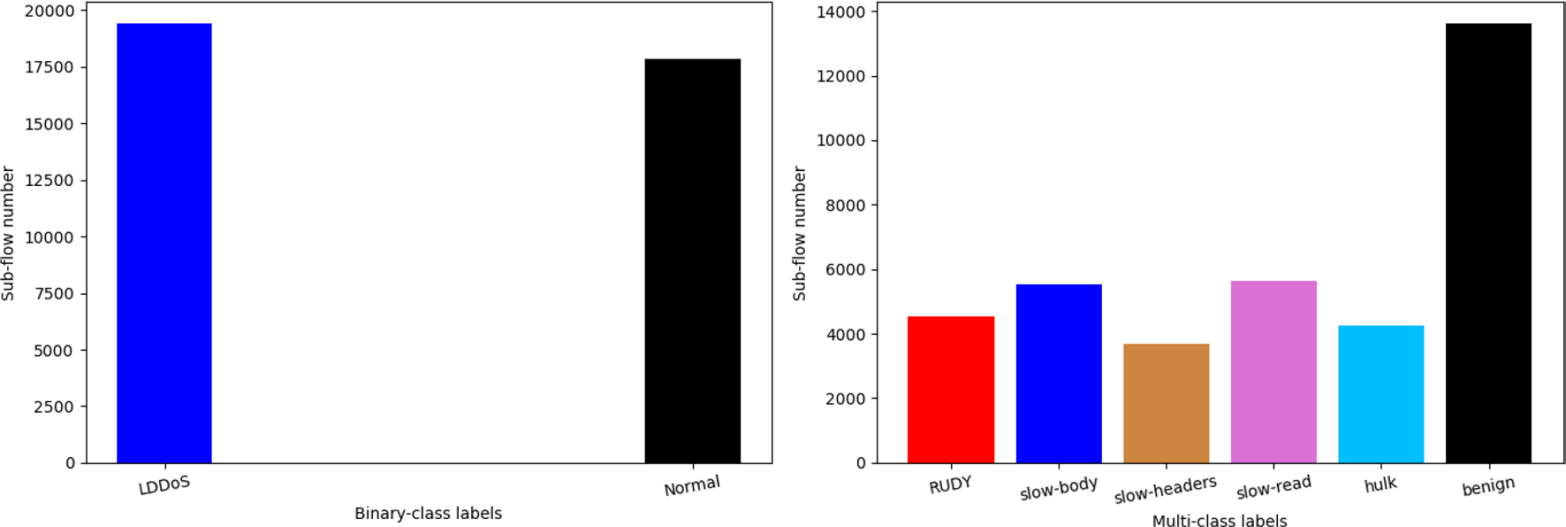
Label distribution on the revised ISCX-2016-SlowDos dataset.

After above data operations, we analyze about whether the data is balanced. As shown in Fig. 6, the numbers of LDDoS and normal sub-flows almost equal in the binary-class scenario. And the numbers of different LDDoS attacks are between 3600 and 5650 in multi-class scenario, they are good for training and testing. Thus, we also down sample the benign number to 5000 during multi-class experiments.

### Experimental setup and evaluation indicators

**Figure 7:**
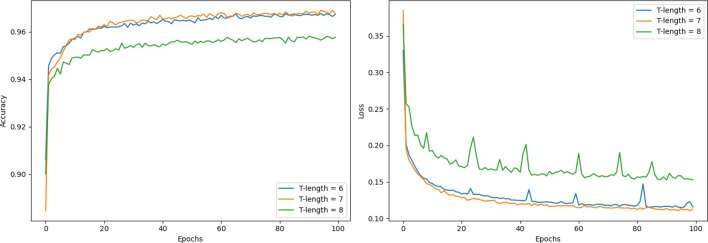
Optimization of fixed *T*-length sliding window.

The experiments are conducted on an Ubuntu deep learning server with an Intel(R) Xeon(R) Silver 4216 CPU, 128G RAM and 2 Nvidia GeForce RTX 3080 graphic card. The proposed model is implemented using the Python 3.6.2 and the library Keras with TensorFlow as the backend. The key well-tuned experiment regarding the selection of fixed length *T* is done as Fig. 7 shown. Normally, some kinds of LDDoS attacks based on TCP three-way handshake are finished in 3 or 4 packets. The other kinds of LDDoS attacks such as slow-read attacks may need 7 packets or so. Thus, the well-tuned range of *T* should be from 3 to 8 theoretically. But the good news is the MSCBL-ADN model has a multi-scale CNN network which can extract feature representation among the 5 most recently arrived packets. As a result, the range of *T* shrinks to [6, 7, 8]. The tuning result shows that the accuracy of model looks best when the *T* is set to 7. The accuracy is as high as 96.74%, and its curve is above the others’. At the same time, we know the average flow size is 7 after packets sorted per communication quintuple in Table 1. That is, the size 7 of sliding window can segment most of flows into sub-flows without padding flow-agnostic packets. This reduces the impact of additional operations. Once *T* is greater than or equal to 8, there could be too many flow-agnostic packets in some segmentations to be detected. In other words, even if the largest 5$\times$5 convolutional kernel is used, it cannot get any useful information. Obviously, this may lead to multi-scale CNN network in MSCBL-ADN model failure. Additionally, in the real network, the collection time of packets increases since the model has to wait for the 8th packet before detection. Overall, the chosen and recommended *T* is 7. And the MSCBL-ADN model parameters and settings are shown in Table 2.

**Table 2:** Parameter setting of MSCBL-ADN model.

Parameter types	Value	Parameter types	Value
Batch size	32	*T*-length	7
CNN scale	1$\times$1, 3$\times$3, 5$\times$5	No. of 1$\times$1 kernel	56
No. of 3$\times$3 kernel	28	No. of 5$\times$5 kernel	28
Bi-LSTM neuron units	128	Dropout rate	0.3
No. of MHA	32	Dense blocks	2
Learning rate	0.001	Epoch	100

To evaluate the performance of model, three evaluation metrics (accuracy, precision, and recall rate) based on confusion matrix and one CPU/GPU elapse (time complexity) are adopted. In confusion matrix, it contains *TP*, *FN*, *FP*, *TN* elements. *TP* means the number of normal sub-flows classified as normal traffic, and *FN* means the number of abnormal sub-flows classified as abnormal traffic. *FP* represents the number of normal sub-flows classified as abnormal ones, while *TN* represents the number of abnormal sub-flows classified as normal ones. According to confusion matrix, accuracy, precision, and recall rate are calculated by Eq. (10). Each experiment runs 10 times by StratifiedKFold, the average values of above estimated metrics are used as the results.

10 $$\begin{aligned} \begin{aligned} {\begin{matrix} &{}accuracy=\frac{TP+TN}{TP+TN+FP+FN},\\ &{}precision=\frac{TP}{TP+FP},\\ &{}recallrate=\frac{TP}{TP+FN}. \end{matrix}} \end{aligned} \end{aligned}$$

### Experiments and comparable experiments

#### Experimental results: classification and time indicators

**Figure 8:**
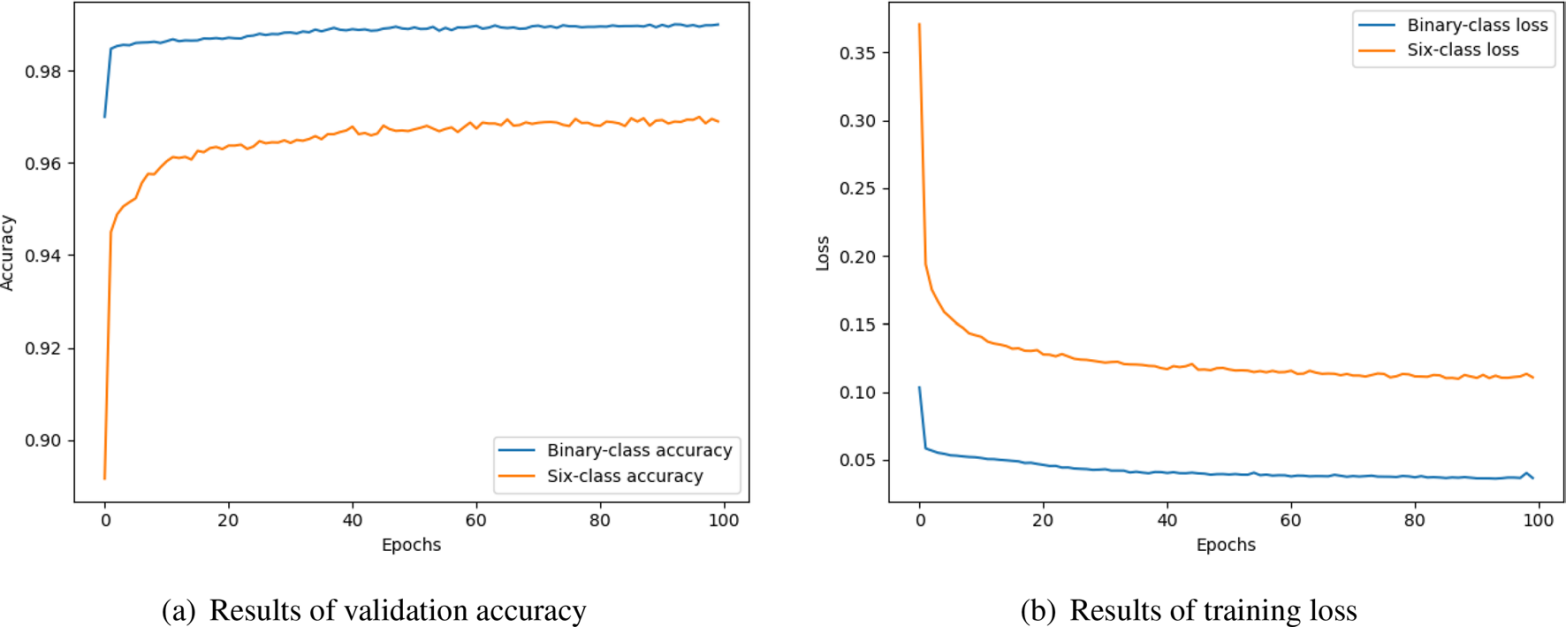
Validation accuracy and training loss of the MSCBL-ADN model.

Figure 8 shows that accuracy and loss curves on the revised ISCX-2016-SlowDos dataset. On the binary-class scenario, the accuracy bumps to 98.53% during first several epochs, and then, increases smoothly and steadily to 98.99%. The loss looks good, too. It decreases gradually to 0.0366. On the multi-class scenario, the accuracy grows quickly from 89.16 to 95.05% in its first four epochs, and increases slowly to 96.90%. At the same time, its loss curve is smooth. The final loss is 0.1108. Obviously, the performance of MSCBL-ADN model is excellent for classifying the network LDDoS attacks. And benefiting from its multi-channel structure, it can be trained a lot of epochs.

**Figure 9:**
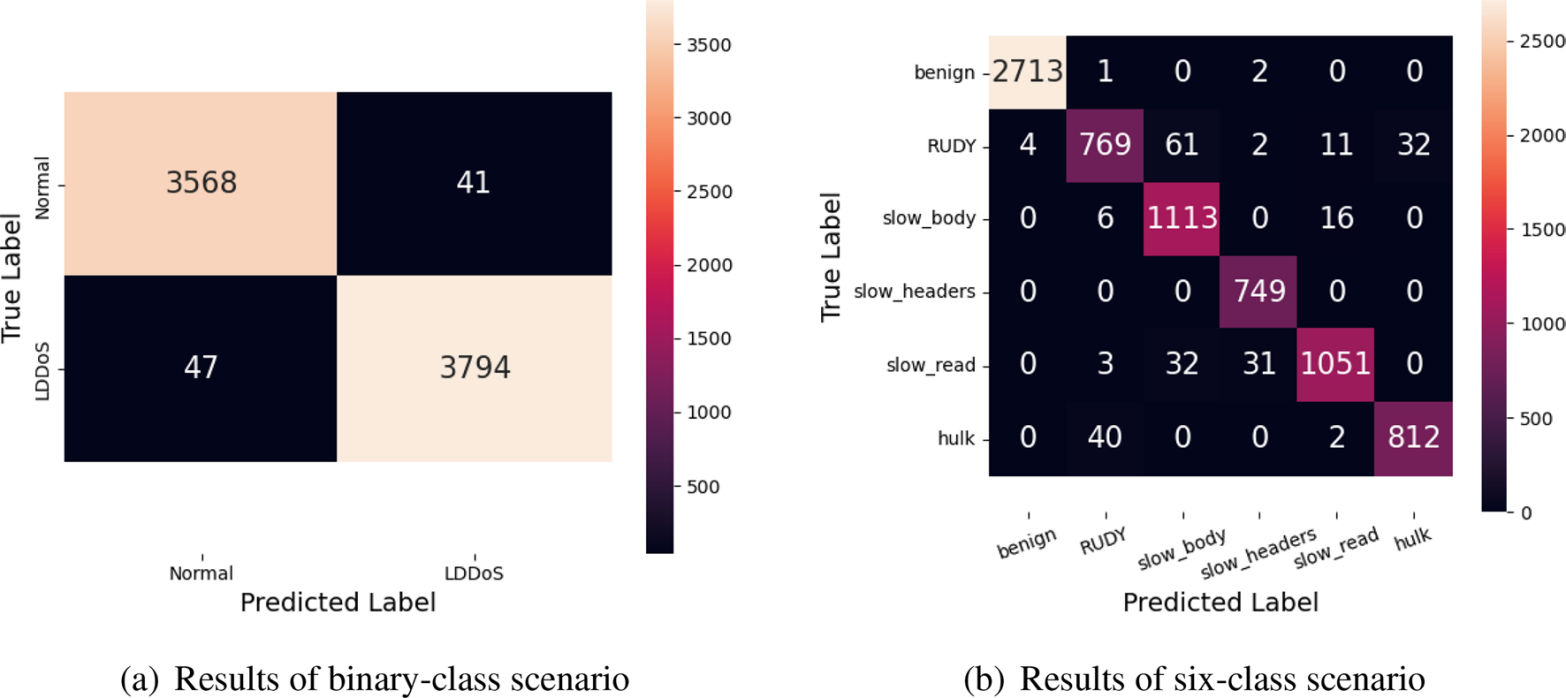
Classification confusion matrix of the MSCBL-ADN model on testing set.

As Figure 9 shown, they are the confusion matrix of classification of MSCBL-ADN model on 20% testing set. Figure 9a is about binary-class scenario. We can get accuracy, precision, and recall rate as 98.78%, 98.82%, and 98.93%. The *FP* and *FN* are both small, which proves the model has good classification ability. Figure 9b is about multi-class scenario. the MSCBL-ADN model has a higher but bearable error rate between RUDY and slow-body. The reason is RUDY is a kind of slow-body fired by RUDY tool. Thus, its features are similar to slow-body ones. The overall accuracy, precision and recall rate are 96.74%, 96.77%, and 96.74%. This indicates the MSCBL-ADN model is good to finish LDDoS attack detection.

The time consumption experiments are run in the mentioned Ubuntu server. The GPU elapse is 5.831 ms every 32 sub-flows. And the time consumption is increased linearly, when we enlarge the number of detecting sub-flows. Thus, the MSCBL-ADN model can be deployed into real network for detection task.

#### Performance comparison experiments

During comparison experiments, we compare the proposed MSCBL-ADN model with RF^18^, SVM^19^ and KNN^20^ shallow classifiers on testing set. We also compare it with CNN-based models (CNN-BMECapSA-RF^25^, LRDADF^27^), LSTM-based models (VLSTM^30^), and CNN-LSTM-based models (AsyncFL-bLAM^16^, NIDS-CNNLSTM^33^) during training and testing phase.

**Figure 10:**
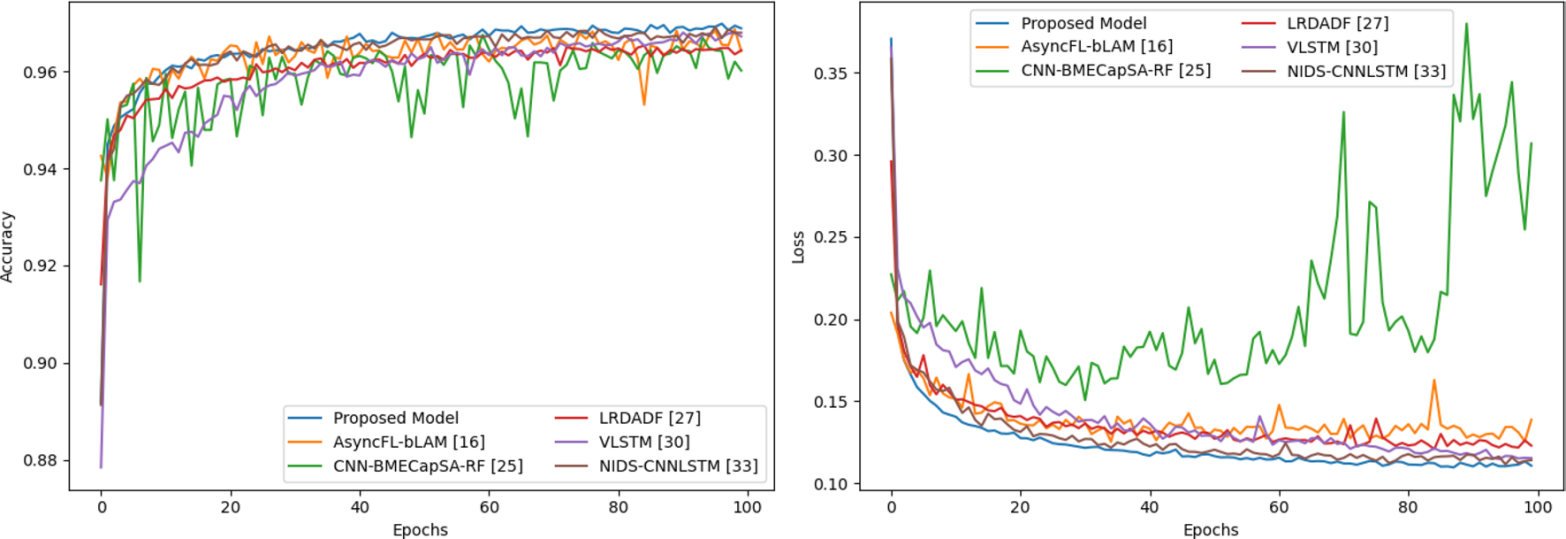
Six-class accuracy and loss comparation during training.

In Fig. 10, it displays the validation accuracy and training loss of multi-class scenario during 100 epochs. We can obviously see that the loss curve of CNN-based CNN-BMECapSA-RF^25^ model is not stable. It explains that the LDDoS attack is strong time relationship, which is a little hard to extract time features to CNN-based model. The LRDADF^27^ model is enhanced by deep sparse autoencoder. As a result, its loss curve is smoother than other CNN-based models. LSTM-based models look better. Their accuracies all can reach to 96% above, and losses are lower than 0.15. The CNN-LSTM-based models improve the accuracies and decrease their losses further. Per the Fig. 10, our proposed model has the best performance.

**Figure 11:**
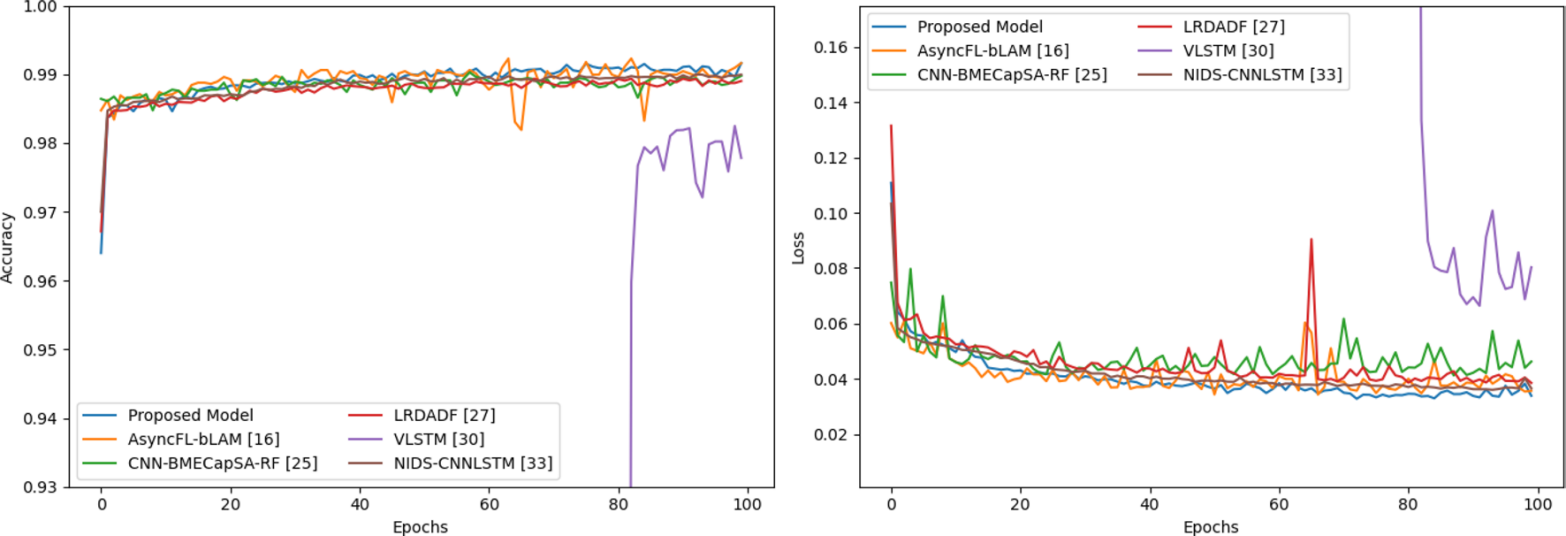
Binary-class accuracy and loss comparation during training.

Figure 11 shows the binary-class training scenario. As known, we merge the hulk and benign samples as normal sub-flows. As a result, VLSTM^30^ model spends lots of epochs to learn time relationship repressions. Until 80th epoch or so, its accuracies and losses are normal. Its first 80 training accuracies and losses don’t show in Fig. 11, since the values are out of the display range. But to CNN-based models, their accuracy curves are graceful. It proves that sparse features are important to detect LDDoS attacks, too. Obviously, the proposed model is a little better than other comparison models. It has an accuracy of 99.04% and a loss of 0.0057 on training set.

The shallow machine learning classifiers have no ability to analyze time steps. Thus, we treat a single sub-flow as a training/testing example. As listed in Table 3, we compare the proposed model with classic machine learning classifiers and deep learning models on testing set on multi-class scenario. The experimental results show that the key indicators of RF^18^, SVM^19^ and KNN^20^ are significantly lower than the deep learning models. But their detection time is faster than deep learning models, since they have only a few parameters for the purpose of classification. Among the deep learning models, the CNN-based models have faster detection speed, but lower detection accuracy. On the contrary, the detection speed of LSTM-based models are slower. In CNN-LSTM-based models, such as AsyncFL-bLAM^16^, NIDS-CNNLSTM^33^, researchers enhanced LSTM part. Their accuracy and detection time gain a little improvement. The testing results demonstrate that the performance of MSCBL-ADN model is a little better than other state-of-the-art models, whose accuracy is as high as 96.74 % and detection time is as low as 5.831 ms each batch.

**Table 3:** Six-class performance comparation on testing set.

Models	Accuracy (%)	Precision (%)	Recall rate (%)	Detection time (ms/batch)
RF^18^	80.48	86.10	73.59	0.872
SVM^19^	84.86	85.81	83.37	0.907
KNN^20^	87.18	87.06	87.18	0.946
CNN-BMECapSA-RF^25^	95.91	94.95	94.69	4.815
LRDADF^27^	95.96	96.01	95.96	5.530
VLSTM^30^	96.01	94.93	94.81	111.438
AsyncFL-bLAM^16^	96.62	95.74	95.64	9.816
NIDS-CNNLSTM^33^	96.44	96.45	96.44	6.867
Proposed model	96.74	96.77	96.74	5.831

## Discussion

Per the results of experiments and comparison experiments, the proposed MSCBL-ADN model has a high accuracy, precision, recall rate, and an acceptable detection time. This benefits from data augmentation method, multi-scale CNN network, time delta embedding-based bi-LSTM network, and multi-head attention arbitration network. Let’s discuss them by ablation studies.

**Figure 12:**
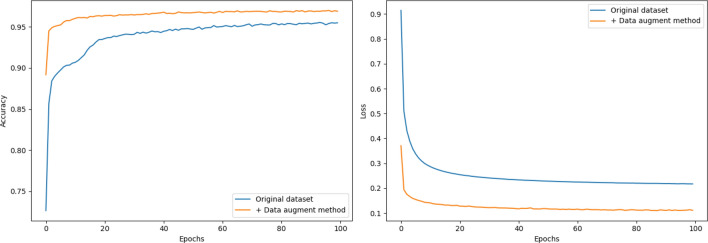
Accuracy and loss comparation w/wo data augmentation method.

The first experiment is used to verify the effectiveness of the fixed *T*-length sliding segmentation data augmentation method. It is conducted with all modules on the original dataset and augmented dataset. As shown in Fig. 12, on the original poor data, the accuracy and loss of MSCBL-ADN model are both impacted. Its final accuracy is lower than 95%, and it has a higher loss as 0.26. When we augment the original dataset, the training data is dilated almost by 10 times. The new augmented dataset is good for training model, since its accuracy and loss curves are both growing steadily. This experiment explains the proposed data augmentation method is effective.

**Figure 13:**
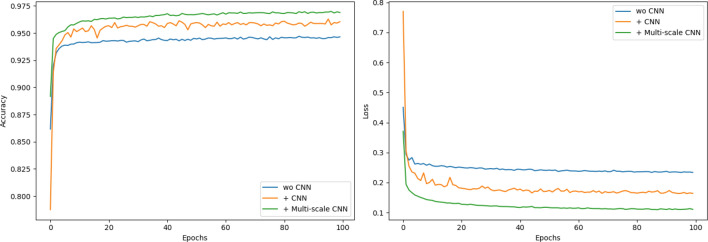
Accuracy and loss comparation w/wo CNN network.

The experimental results shown in Fig. 13 are about the effectiveness of multi-scale CNN network. We conduct the experiments without CNN network, with CNN network and with multi-scale CNN network. Without CNN network, the proposed model can learn how to detect LDDoS attacks, but the accuracy is low. By using CNN network, the sparse representation can be learned for detection. As known, some LDDoS attacks, such as slow-read attacks, need more than one packets for firing an attack. That is, we need different scale CNN kernels for learning representations among packets. As a result, multi-scale CNN network model has the best accuracy and the smallest loss.

**Figure 14:**
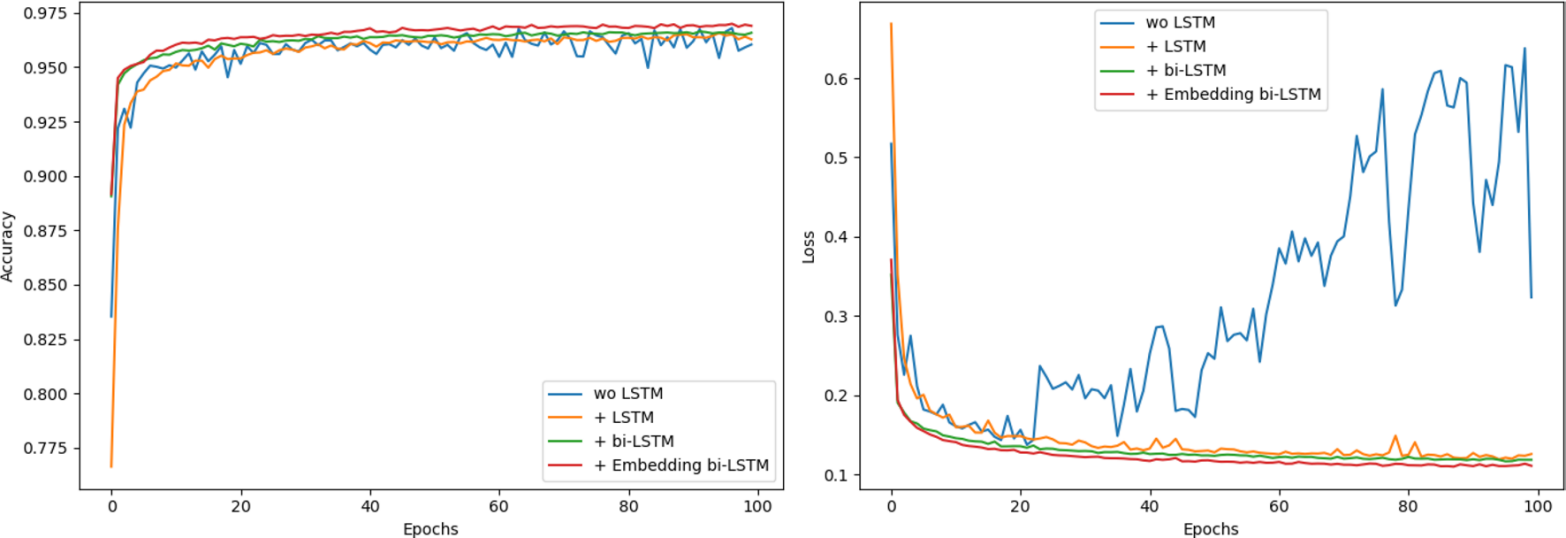
Accuracy and loss comparation w/wo LSTM network.

The basic idea of third group of experiments is to verify time delta embedding-based bi-LSTM network. We compare the accuracies and losses in the experiments without LSTM network, with LSTM network, with bi-LSTM network and with embedding-based bi-LSTM network. The packets of LDDoS attacks have strong time positive correlation. As shown in Fig. 14, without LSTM network, the accuracy curve is volatile, and its loss curve is growing after dozens of epochs. With LSTM network, the curves look better. By using bi-LSTM network, the model can learn from both the front and back packets. This can alleviate the bad impact of missed packets in sub-flows, and improve the accuracy further. When we embed the time delta info between packets into bi-LSTM network, the model is enhanced. From the experimental outputs, our proposed network is more accurate and effective.

**Figure 15:**
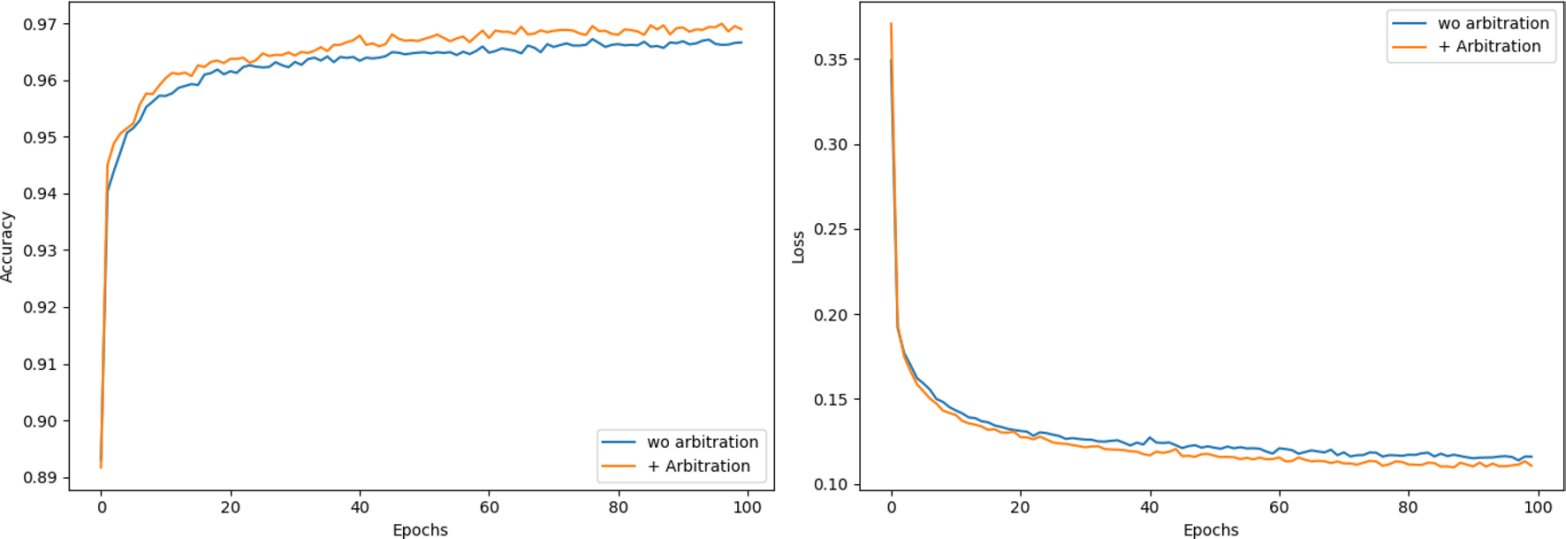
Accuracy and loss comparation w/wo arbitration network.

The arbitration network helps a lot, too. The representations from multi-scale CNN network and embedding-based bi-LSTM network are extracted parallelly and independently. Thus, an arbitration network is needed to decide which representations are key items. As illustrated in Fig. 15, the performance is better with it.

For saving the detection time, benefiting from the fixed *T*-length sliding segmentation method and padding technology, when any packet comes, the LDDoS detection can start. This speeds the frequency of detection up in real network world. In bi-LSTM network part, we only allow time related features as inputs. Thus, the number of bi-LSTM neurons can be small, which saves the scale of training parameters and speed detection up. The MHA structure handles the inputted representations parallelly. This makes use of multi-GPU processors and saves time. By above technologies and tricks, the MSCBL-ADN model gets LDDoS detection results in an acceptable amount of computational time.

## Conclusions

LDDoS attacks are a kind of new worldwide network security issues. There is only a little poor public dataset used for training AI model currently. In this paper, we have designed and verified an effective LDDoS attack detection model under the limit of poor data, called MSCBL-ADN. It augments the dataset by a novel fixed *T*-length sliding segmentation data augmentation method. It incorporates multi-scale CNN and embedding-based bi-LSTM for key feature extraction, and employs arbitration network for redistributing weights of key representations. The experimental results demonstrate that MSCBL-ADN model has good performance in both binary-class and multi-class scenario. In the near future, we wish to enhance the MSCBL-ADN model with two tasks. The task is to try and replace LSTM-like network with attention network for the purpose of speeding up detection time further. At the same time, we also find there is a high error rate between hulk and slow-body attacks per experimental results. Thus, the other task is to analyze the detailed reason and retrain the enhanced MSCBL-ADN model.

## Data Availability

The datasets analysed during the current study are available in the Canadian Institute for Cybersecurity webpage https://www.unb.ca/cic/datasets/dos-dataset.html, and in the MIT webpage http://www.ll.mit.edu/r-d/datasets/1999-darpa-intrusion-detection-evaluation-dataset.
